# Gender differences in smoking cessation support: a qualitative study of Dutch healthcare professionals’ experiences

**DOI:** 10.1093/heapro/daaf183

**Published:** 2025-11-10

**Authors:** Fien M van de Kamer, Judith E M Visser, Andrea D Rozema, Anton E Kunst, Irene G M van Valkengoed, Mirte A G Kuipers

**Affiliations:** Amsterdam UMC, University of Amsterdam, Department of Public and Occupational Health, Amsterdam Public Health Research Institute, University of Amsterdam, Van der Boechorststraat 7, 1081 HV Amsterdam, The Netherlands; Amsterdam UMC, University of Amsterdam, Department of Public and Occupational Health, Amsterdam Public Health Research Institute, University of Amsterdam, Van der Boechorststraat 7, 1081 HV Amsterdam, The Netherlands; Tranzo Scientific Center for Care and Wellbeing, Tilburg School of Social and Behavioral Sciences, Tilburg University, Professor Cobbenhagenlaan 125, 5037 DB Tilburg, The Netherlands; Amsterdam UMC, University of Amsterdam, Department of Public and Occupational Health, Amsterdam Public Health Research Institute, University of Amsterdam, Van der Boechorststraat 7, 1081 HV Amsterdam, The Netherlands; Amsterdam UMC, University of Amsterdam, Department of Public and Occupational Health, Amsterdam Public Health Research Institute, University of Amsterdam, Van der Boechorststraat 7, 1081 HV Amsterdam, The Netherlands; Amsterdam UMC, University of Amsterdam, Department of Public and Occupational Health, Amsterdam Public Health Research Institute, University of Amsterdam, Van der Boechorststraat 7, 1081 HV Amsterdam, The Netherlands

**Keywords:** gender, smoking cessation, professionals’ support, professionals’ experiences, qualitative research

## Abstract

Effectiveness of smoking cessation support differs between men and women. In order to gain a comprehensive understanding of the gender differences, this study aimed to investigate the experiences of professionals providing support, focusing on perceived variations by clients’ and professionals’ gender. In 2024, qualitative semi-structured interviews were conducted with 15 professionals providing smoking cessation support in the Netherlands. Interviews were transcribed and analyzed using an inductive reflexive thematic approach. Gender differences were categorized into themes related to the clients’ and professionals’ gender. Identified themes related to clients’ gender were the following: openness to support (e.g. men more often show resistance to support in contrast to women who are generally more open to support), type of support needed (e.g. men more often need structured support), clients’ attitude in the support (e.g. men more often have a pragmatic attitude, women are more likely to talk about feelings), and interaction between client and professional (e.g. men should be challenged more and women should be encouraged in their quitting process). Themes related to the professionals’ gender were: professionals’ portrayal in the support (e.g. professional men are more likely to project authority, professional women are often more easily accessible) and interaction between professional and client (e.g. professional men are more likely to communicate more directly, professional women are more likely to be gentle). The differences suggest that the gender of both clients and professionals may influence the support provided, highlighting the importance of taking gender differences into account when providing smoking cessation support.

Contribution to Health Promotion

- Professionals experience gender differences in smoking cessation support, particularly in clients’ openness, support needs, attitudes, and interaction dynamics.
- Professionals perceive their own gender as having little influence on smoking cessation support, reflecting only minor differences between professionals of different genders.
- By exploring professionals’ experiences, the study highlights that gender differences may influence the smoking cessation support provided, suggesting the importance of taking gender into consideration when providing support.
- The study findings may help understand gender differences in smoking cessation intervention effectiveness and suggest ways for professionals to improve their support.

## BACKGROUND

The prevalence of smoking worldwide remains high, with 1.3 billion tobacco users, comprising of 37% of men and 8% of women (WHO 2023). This tobacco epidemic is one of the main public health threats the world faces, killing over 8 million people globally each year (WHO 2023). In the Netherlands, the prevalence of cigarette smoking stabilized at 19% in 2022 and 2023, with a prevalence of 15% among women and 23% among men (RIVM 2023). Gender, alongside factors such as socio-economic position and ethnicity, is an important determinant of smoking behavior (Bolego *et al*. 2002). Even though women who smoke daily tend to smoke fewer cigarettes per day and have lower nicotine dependance (Allen *et al*. 2016), men are more successful at quitting than women and are more likely to remain abstinent in the long-term (Smith *et al*. 2016). To increase equity in smoking cessation outcomes, it is important to gain a better understanding of gender differences in smoking cessation.

A body of literature has studied the experiences and needs of men and women in smoking cessation. Studies found that men and women experience different barriers when attempting to quit smoking or accessing support (Copeland *et al*. 2010, Dieleman *et al*. 2021, Thompson *et al*. 2015). For example, women more often than men experience barriers for smoking cessation in emotional and stressful events, the need to adhere to societal beauty standards (e.g. preventing weight gain), mental health disorders and absence of childcare services (Minian *et al*. 2016). In contrast, men report facing barriers related to external factors such as the high availability of tobacco products, social activities where smoking is prevalent and alcohol consumption going hand in hand with smoking (Dieleman *et al*. 2021, Torchalla *et al*. 2011), but also a belief that quitting is impossible or depends solely on their own determination (Van Do *et al*. 2020). Studies on the needs of men and women in smoking cessation found that men mainly favor structured and intensive programs, employing 24/7 buddy systems and reward-based therapies, while women mostly prefer non-nicotine behavioral group interventions characterized by emotional assistance, positivity, and empathetic communication (Allen *et al*. 2014, Dieleman *et al*. 2021).

Despite these insights, the perspectives of professionals remain understudied, while their role will be crucial in delivering smoking cessation support in a gender-responsive manner. Understanding how professionals in smoking cessation services acknowledge and experience these gender-specific challenges is crucial in improving the success of interventions. Moreover, there is a lack of understanding regarding how professionals perceive the role of their own gender in smoking cessation support, while smoking cessation counseling practice may also vary between professionals who are either men or women.

The aim of this study was to investigate the experiences of professionals providing smoking cessation support, focusing on professionals’ perceived differences in both clients’ and professionals’ gender.

## METHODS

### Study design and setting

An interpretative qualitative research design was applied (Alase 2017). The study was conducted in the Netherlands. In the Netherlands, a smoking cessation program typically includes behavioral counseling—either in a group or individually; face-to-face, online, or by telephone—in combination with pharmacological options such as nicotine replacement therapy (NRT) (Willemsen *et al*. 2013). Smoking cessation support (including behavioral and pharmacological support) is reimbursed by Dutch health care insurance providers once a year, without out-of-pocket costs.

### Study population and recruitment

Participants were included in the study if they were (1) professionals who provided smoking cessation support, part-time or full-time, employed by private or public organizations, or worked independently; (2) working in the Netherlands; and (3) in direct contact with patients. Professionals were excluded if they were (1) not able to understand or speak Dutch; and (2) specialists providing secondary and tertiary care, such as pulmonologists, and professionals providing specialized mental health services.

We recruited participants using purposive sampling to achieve a heterogeneous study population. First, we reached out to an existing network of different professionals across various organizations, such as general practices, smoking cessation services and social and community organizations. Second, we reached out to other general practices and smoking cessation organizations with whom we had no prior contact. Last, participants were also recruited by snowball sampling, through referrals from the previously recruited participants (Parker *et al*. 2019). In total, 28 potential participants were approached and 15 participated. Reasons for not participating included a lack of interest or insufficient time. To ensure sufficient representation, an as equal as possible number of people who identified as men and women were recruited.

### Conceptualization of gender

In this study, we focus on gender differences rather than biological sex differences, as our interest lies in how social factors related to gender shape the experiences of people with smoking cessation support (Fausto-Sterling 2019, Greaves *et al*. 2020). Gender is conceptualized as the power relations, norms and expectations of women, men and gender-diverse people that shape how individuals view themselves and each other, how they behave and interact, and reflect how power is distributed in society (Fausto-Sterling 2019, Greaves *et al*. 2020). We therefore reflect from this perspective. Even though we acknowledge the broad range of gender identities, this study concentrated exclusively on people who identify themselves as men or women, as these are the two largest gender groups, and in order to limit the scope and enhance the focus of the study.

### Data collection

F.v.d.K. conducted 15 in-depth semi-structured interviews from March to May 2024. Before the interviews, participants were provided with an information letter and an informed consent form. A short demographic questionnaire was also conducted prior to the interview, consisting of seven questions on characteristics such as the sex, gender identity and work experience of the professional. Interviews were conducted at a location chosen by the participant (N = 4) or online via Microsoft Teams (N = 11), depending on the participant’s preference. The length of the interviews varied from 32 to 60 minutes. All interviews were audio-recorded via a voice recorder and field notes consisting of notable remarks were taken by the researcher during the interviews. After each interview, a member check was sent to the participant, consisting of a short summary of the data collected, developed by F.v.d.K. Participants could then verify the accuracy of the data and provide additional input if necessary (Braun and Clarke 2023b ).

### Instruments

To structure the interview guide, the transtheoretical model of change (TTM) was used (see Supplementary material 1). The TTM helped facilitate the conversation about the participant’s perceived gender differences in four of the model’s stages of behavior change: contemplation (i.e. starts thinking about behavior change), preparation (i.e. preparing for behavior change), action (i.e. performing the desired behavior), and maintenance (i.e. maintaining the behavior long-term without relapse) (Armitage 2009, Prochaska and Velicer 1997, Robinson and Vail 2012). We omitted the precontemplation stage from our interview guide, as professionals are not primarily involved in this phase.

For each of the four TTM stages the interviewer asked about the experiences of the professional, after which the interviewer asked in-depth questions regarding clients’ and professionals’ gender. For example, questions about the contemplation phase included were the following: “To what extent is the support you provide as a professional accessible to everyone?” “To what extent do you observe differences between men and women?” and “To what extent do you think your gender (being a man/woman) influences the accessibility?”.

### Data analysis

We applied reflexive thematic analysis using an inductive approach (Braun and Clarke 2023a , Mortelmans 2020) to describe and summarize findings and afterwards interpret these findings (Braun and Clarke 2023a, 2023b). The analysis followed an iterative process, in which we followed the phases of thematic analysis according to Braun and Clarke (Braun and Clarke 2006). The coding process was carried out in MAXQDA (version 22.1.1).

First, the data gained from the audio-recorded interviews were transcribed verbatim after which the transcripts were read. Second, F.v.d.K. generated initial codes, starting by labeling relevant excerpts containing information relevant to the aim of the study. The first two transcripts were independently doubled-coded by two researchers (F.v.d.K. and J.V.). We compared the codes and discussed inconsistencies among three researchers (F.v.d.K., J.V., and M.K.), and codes were adjusted. Third, after coding all transcripts, the first and second authors (F.v.d.K. and J.V.) divided all codes into two categories: codes related to the gender of the professionals and codes related to the gender of the clients. Next, the codes were grouped to form broader themes, respectively for gender of the client and for gender of the professional. Fourth, in discussions between F.v.d.K., J.V., and M.K., these themes were reviewed and refined so that the themes fully represented the data. Fifth, F.v.d.K. identified subthemes and F.v.d.K., J.V., and M.K. discussed and refined the names and contents of each subtheme. This led to main themes and subthemes, separately related to the gender of clients or professionals (Braun and Clarke 2006).

We also examined whether the themes that emerged from the contributions of professionals differed between men and women. However, the analysis showed that all identified themes came from both genders, indicating that there was no gender-specific variation in the thematic structure of the data.

### Reflectivity

The researchers responsible for recruiting participants, conducting interviews, and analyzing the data, F.v.d.K. and J.V., are both *cis*-women, born in the Netherlands, non-religious and non-smokers. We therefore recognize that our personal identities and lived experiences influence our perspectives on smoking cessation and gender. We did not have specific prior conceptions of gender in the context of smoking cessation, being relatively new to the topic. Nonetheless, we were aware of our position as women and recognized that this could influence our interactions with participants and interpretations of the findings. Throughout the research process, we remained attentive to these considerations and aimed to stay close to the participants’ lived experiences. To ensure reflexivity in the qualitative analysis, we engaged in regular discussions with other researchers, including men, and with researchers who had expertise in gender studies, in order to critically examine potential biases.

### Ethical Statement

The Medical Ethics Review Committee of the Amsterdam UMC confirmed that the Dutch Medical Research Involving Human Subjects Act (WMO) did not apply to this study and that official approval was not required (reference number 2023.0957).

## RESULTS

### Participant characteristics

Background characteristics of the participants are provided in Table 1. Participants’ age ranged from 24 to 66 years, with an average of 53 years. All participants were *cis* gender; eight women and seven men were interviewed. Most participants worked as smoking cessation specialists (i.e. providing smoking cessation support as their primary role), two participants were general practice nurses and one participant was a lifestyle coach (both providing smoking cessation support as part of their broader responsibilities). Most participants had five or more years work experience in smoking cessation.

**Table 1.** Background characteristics of the participants.

		N	%
Total		15	100
Sex			
	Men	7	47
	Women	8	53
Gender			
	Men	7	47
	Women	8	53
Age			
	18–29	1	7
	30–39	1	7
	40–49	1	7
	50–59	7	47
	60–69	5	33
Working setting			
	General practice	3	20
	Smoking cessation organization	9	60
	Hospital	3	20
Work experience in smoking cessation			
	1 or 2 years	3	20
	3 or 4 years	1	7
	5 or more	11	73

### Differences related to the client’s gender

Various perceived differences were identified related to clients’ gender, which are categorized into four themes (Table 2): openness to support, type of support needed, client’s attitude in the support and interaction between professional and client.

**Table 2.** Main themes and subthemes—differences related to the client’s gender in smoking cessation support according to professionals themselves.

Main themes	Subthemes	
	Clients who are men	Clients who are women
**Theme 1: Openness to support**	1.1 Men have a passive approach to professional help 1.2 Men show resistance to support	1.3 Women are more open to support
**Theme 2: Type of support needed**	2.1 Men need more assistance in the support 2.2 Professionals discuss with men how to deal with social pressure at work 2.3 Professionals guide men to sport activities as a coping strategy of smoking	2.4 Women need social cohesion in the support 2.5 Women need to discuss women’s issues (pregnancy, menopause) 2.6 Professionals guide women to creative activities as coping strategy of smoking
**Theme 3: Client’s attitude in the support**	3.1 Men show dominant/macho behavior in the support 3.2 Men are not vulnerable/maintain a pragmatic attitude	3.3 Women talk more about feelings, focus more on emotions during the support 3.4 Women who smoke secretly are less open about relapse behavior 3.5 Women exhibit an active stance in the support
**Theme 4: Interaction between professional and client**	4.1 Men need to be triggered 4.2 Men need straight forward communication 4.3 Power dynamics between professional and client can lead to boundary violations	4.4 Women need encouraging communication

#### Theme 1: openness to support

Professionals experienced that men generally show more resistance to support provided by a professional, expressing their desire to act independently. Professionals reported to see many clients who were men expressing a preference for quitting smoking on their own, with minimal external assistance. *“Men often say: ‘I will do it myself, I’ll read the book and fill in the cessation plan. No, I just must be able to do it you know, I think that is all nonsense’.”—(woman, 52).* This resistance was closely related to the higher dropout rates observed by professionals among clients who were men. These clients were also less likely to return for support after a relapse, which professionals attributed to feelings of shame or embarrassment about their failure.

When men participated in smoking cessation support, professionals were more likely to observe a more passive approach. This was characterized by relying on nicotine patches and medication, instead of attending the sessions and doing exercises at home. They often prioritized other appointments, such as work commitments, over attending the smoking cessation sessions. This necessitated a more proactive role from the professional. They reported the necessity to provide extensive explanations and to activate the intrinsic motivation of their clients who were men.

In contrast, professionals perceived that women tend to be more open and engaged in the smoking cessation support process. According to professionals, women were more likely to seek help and actively participate, also outside of the consultations. Subsequently, their proactive attitude made them more accessible. *“Women are like: ‘well, I want to stop and I want help’.” —(woman, 53).*

#### Theme 2: type of support needed

Professionals noted that in smoking cessation support, they provide men with more guidance throughout the support than women, including clearer and more concrete explanations and assigned tasks to keep them motivated and engaged. *“The no-nonsense people, or those who expect a bit more of a non-nonsense attitude from themselves [say]: ‘stop complaining and just do it’, directed at themselves. Which does not work. And if we then recognize that, you know, the brain is sometimes a bit more complex than that, this gives more recognition and a bit more support, especially for men.”—(woman, 52).*

In addition, professionals indicated that they more often discuss with men than women how to deal with social pressure at work, as this was a major pitfall for returning to smoking. According to professionals, the way men interact with their peers in the workplace encouraged smoking due to social pressure from colleagues. In addition, social pressure was also present during other social activities. However, when these clients were unable to deal with these challenges, professionals found it difficult to re-motivate them. *“With men, you often see them drinking a beer at the pub, playing billiards, or darts on the weekends. They think: I can take one [cigarette], but that's just not possible. Oh well, they need to learn all that.”—(woman, 53).*

Many of the professionals actively involved the social environment of the client in their support, with clients who were women generally having a greater intrinsic need for the involvement of social relations compared to men. Moreover, professionals also observed that women were more likely to seek support from other women during group sessions. *“Women are more likely to involve a partner or involve someone from their social environment or a sister or friend.”—(man, 56).*

Most professionals mentioned that they consider biological factors, particularly in the support they provide to women. For example, professionals accounted for menopause because smoking withdrawal symptoms can mimic menopausal symptoms. Additionally, for pregnant women or women who want to become pregnant, professionals increased the number and duration of consultations.

Outside of the support sessions, coping strategies for smoking cravings were a frequently mentioned as effective strategy to help clients manage their smoking behavior and relapse. As a diversion, several professionals led clients who were women toward creative pursuits, such as knitting, crocheting and diamond painting. However, these activities, which women mentioned as (former) hobbies, unintentionally leaned toward more traditionally feminine activities. On the other hand, men rarely reported these creative activities, and professionals often guided them toward sport activities such as playing darts and walking instead.

#### Theme 3: client’s attitude in the support

Professionals highlighted that men maintained a pragmatic mindset during the support, characterized by an aversion to discussing their smoking behavior or discussing their thoughts, especially in group support settings. Their attitude toward smoking cessation was generally straightforward, “just stop smoking” without wanting to delve deeper into their feelings, emotions and underlying issues. *“I think that for some men it is less, perhaps also a kind of certain societal belief that you must not talk about your feelings.”—(woman, 31). “Yes, to show more of the emotional side of what’s happening, especially with men. Yes of course, not all men, but in general, I think it is more difficult for men to talk about it, and it would be better to focus on what he thinks rather than what he feels.”—(woman, 55).*

Moreover, professionals reported that some men showed dominant and macho behavior when approached by the professional, making them less approachable. Some professionals mentioned that men could place themselves above the professional, expecting the professionals to listen to them, instead of the professional being in charge. This could lead to a tense atmosphere between the client and the professional.

In contrast, professionals experienced clients who were women to have a more welcoming attitude, in which they often presented a more vulnerable side of themselves during the consultations. They were more likely to be open about their feelings, frequently discussing their emotions such as loneliness and personal challenges during the support. For example, they more often shared personal challenges such as burnout, psychological or familial problems as contributing factors to their smoking behavior. As a result, professionals generally experienced a more challenging progression of smoking cessation among these clients. *“When it comes to women, they are more inclined to say something like: ‘the situation was incredibly stressful I did not know what to do’.”—(woman, 53).* Conversely, professionals emphasized that women who smoked in secret were less open than men about their relapse behavior, often due to the shame of not fitting the role model of a mother in front of their families.

In addition to women being more open about their feelings, professionals also observed that women tended to adopt an active attitude and reflective approach, in which women showed empathy toward the professional and independently searched for effective methods to quit smoking. A professional noted that this aligned with society’s expectations, where women are often portrayed as emotionally expressive and service-oriented. *“I think in general that women are more inclined to talk to me about things, so we quickly pick up on a topic and then, from their own experience, they might tell something or recognize it and say it, or mention that it happened to their neighbor or something like that.”—(man, 56).*

#### Theme 4: interaction between professional and client

Professionals reported that clients who were men often needed to be triggered and convinced to quit smoking. In addition, professionals often prefer a more explicit and straight to the point form of communication and interaction with men. *“When you speak to a man, you have to challenge him: ‘Hey, are you sure you can manage this without it?’ Frequently, they need to be convinced, but once convinced they go for it.”—(man, 61)*. In clients who were women, professionals experienced that a more encouraging communication style generally works best. *“If women feel completely confident, then they say: ‘that is what I am aiming for.’ So, then you must give them trust. While I think with men you should challenge them more.”—(man, 61).*

A couple of professional women mentioned that the interaction between them and clients who were men occasionally led to transgressive behavior from these clients. These behaviors ranged from inappropriate invitations to social activities, requests for appointments outside of the consultation sessions and inappropriate physical contact. Consequently, these professionals integrated other activities in their support to minimize the physical contact and adjusted their communication in such situations. *“Asking me out for dinner or trying to touch me all the time. That is a possibility, so if you look at such behavior, I do not experience that with women. These are only men who might find that interesting.”—(woman, 31).*

### Differences related to the professional’s gender

Although according to most professionals the role of their own gender was small, some differences were identified related the professionals’ gender, which are categorized into two themes (Table 3): professional portrayal in the support and interaction between gender professional and client.

**Table 3.** Main themes and subthemes—differences related to professional’s gender in smoking cessation support according to professionals themselves.

Main themes	Subthemes	
	Professional men	Professional women
**Theme 1: Professional portrayal in the support**	1.1 Professional men are more likely to project authority toward their clients 1.2 Professional men are seen as doctors	1.3 Professional women are more easily accessible for support and relapse recovery
**Theme 2: Interaction between gender professional and client**	2.1 Clients who are men prefer interaction with men professional 2.2 Professional men use a more direct and strict communication styles 2.3 Professional men are less likely to discuss personal problems in detail	2.4 Clients who are women prefer interaction with professionals of the same gender 2.5 Professional women use less direct and intimidating communication style 2.6 Professional women are more likely to delve deep into personal problems

#### Theme 1: professionals portrayal in the support

Some professional men indicated that being a man may project more authority toward their clients, in particular toward women. Moreover, professional men were often automatically assumed by both men and women to be doctors. *“You can see a societal trend where men are often slightly more respected and occupy higher positions. That is just unconscious for a lot of people.”—(man, 24).*

Participants indicated that access to support and return after relapse was considered easier when a professional woman was involved. *“A colleague mentioned to me this afternoon: ‘Hey, you are so sweet and petite, which can make you less intimidating for men.’ And it is easier to come back compared to sitting opposite a man where they might feel more vulnerable.”—(woman, 31).*

#### Theme 2: interaction between gender professional and client

While most professionals have not received feedback from clients regarding a gender preference of the professional, some professionals believed that their clients might prefer receiving support from professionals with a similar gender identity. *“Well, I can imagine that women might feel a bit safer with a woman [professional].”—(woman, 46).*

In addition, professionals indicated that their style of communication depended on their gender. Professional men reported that they used a direct and strict communication style toward their clients, especially men due to the gender congruence. *“So, my own gender, well is a bit influential in that I tell those guys: ‘you know if you do this or that, that it is not cool, man. Come on, show that you are a man’, that kind of conversation.”—(man, 54).*

Professional women noted that they tend to have a gentler approach in communication, daring to ask more emotional questions and use a less direct, strict and intimidating tone toward their clients. *“I am not like that. Sure, sometimes I feel I could have been a bit more straightforward or assertive, but it is simply not part of my nature. Men on the other hand, might have a more direct approach or use humor, which I might lack.”—(woman, 52).* In addition, with clients who faced multiple problems, professional men tended not to discuss these problems in detail, while professional women tended to delve into these topics more extensively.

## DISCUSSION

### Key findings

This study explored the experiences of professionals providing smoking cessation support, focusing on perceived differences by both clients’ and professionals’ gender. Several themes were identified related to clients’ gender: openness to support (e.g. men more often show resistance to support in contrast to women who are generally more open to support), type of support needed (e.g. men more often need structured support), clients’ attitude in the support (e.g. women are more likely to talk about feelings) and interaction between client and professional (e.g. men should be challenged more often and women should be encouraged in their quitting process), and related to professionals’ gender: professionals’ portrayal in the support (e.g. professional men are more likely to project authority, professional women are often more easily accessible) and interaction between professional and client (e.g. professional men are more likely to communicate more directly, professional women are more likely to be gentle).

### Interpretation of the findings

The findings reveal gender differences in clients’ openness to professionals’ support, based on the experiences of professionals, with men more often showing a more passive approach compared to women. One explanation could be that men have been found to be generally less concerned with health-related problems and therefore more often tend to resist seeking support when they need help (Bottorff *et al*. 2006, Courtenay 2000, Emslie and Hunt 2008). Moreover, previous literature has highlighted men’s reluctance to receive direct advice from a professional, underscoring their preference for autonomous decision making and self-reliance in their cessation process (Bottorff *et al*. 2018). This preference may be attributed to culturally determined masculine characteristics, particularly the mandate to ‘be strong’, which is reflected in an avoidance of seeking or asking for help (Camacho-Ruiz *et al*. 2024). This can influence the way professionals interact with clients who are men. For example, participants reported that confronting men with their resistance, giving them assigned tasks and challenge them, keeps them more motivated and engaged. For example, incorporating competition into smoking cessation support is seen as highly motivating by men, which could explain why professionals are more likely to challenge men in the support (Bottorff *et al*. 2018). In addition, research showing that qualities such as strength and resilience can be key factors to persuading men that they can quit and possibly maintain their behavior (Bottorff *et al*. 2018, Oliffe *et al*. 2012).

The findings demonstrate that professionals experienced that women are generally more open to support and more active during the support sessions. This is in line with previous research showing that women take an active stance in support and prefer more frequent, additional contact, e.g. through telephone calls (Minian *et al*. 2016). This may be attributed to women being more likely than men to seek help in response to concerns about physical symptoms, psychological problems, or task failure (Sorensen and Pechacek 1987). In addition, women may experience stronger social pressures to quit than men, due to the greater social disapproval (Dohnke *et al*. 2011). For example, women may not only feel societal pressure to quit smoking to live up to the ideal of a good mother or good partner but also experience pressure from these expectations. Similarly, once pregnancy is confirmed, the majority of women try to quit (Buczkowski *et al*. 2014). Traditional gender roles assigned to women therefore affect their smoking cessation trajectory.

The study shows that professionals do not consider the role of their own gender to be significant. However, a broader analysis of the findings shows that their gender does influence their own communication style, the client's behavior, and the expectations of their colleagues, suggesting that professionals’ gender may influence the support provided. For instance, professionals believed that their clients might favor support from someone who shares the same gender identity. Although professionals, both men and women, may be perceived as equally skilled and knowledgeable (Kerssens *et al*. 1997), preferences may be explained by easier and more informal communication between doctors and patients of identical gender identities (van den Brink-Muinen *et al*. 1998). This aligns with earlier research in primary care, which found that patients who are women often feel more comfortable and less embarrassed in communication with doctors with the same gender (Roter *et al*. 1991). Patients who are men who favor health professionals who are men use the similar reasons (Roter *et al*. 1991). This may have significant consequences, as women visiting doctors who are men showed more irritabilities, anxiety, and less friendly behavior (Roter *et al*. 1991).

### Strengths and limitations

This study is among the first to investigate professionals’ experiences providing smoking cessation support, focusing on perceived differences in both clients’ and professionals’ gender. A strength of this study is that we conducted member checks, which confirms that the findings accurately reflect the participant’s experiences and thereby increasing the credibility and confirmability of the study.

Several limitations should, however, be considered. There is a risk of response bias due to social desirability, as participants might withhold information when discussing sensitive topics. Discussions about gender issues can be sensitive, potentially leading professionals to provide socially acceptable answers rather than expressing their exact experiences and perceptions. Second, the interviewer’s repeated focus on gender differences may have unintentionally reinforced stereotypes or caused professionals to downplay gender-related experiences in smoking cessation support. Third, a limitation of this study is the lack of representation from non-cisgender groups. Since these experiences may differ significantly from those of cisgender individuals and were not specifically explored, some relevant data may be missing. There is a need for future studies on experiences with gender minority groups, in order to be able to address the full gender spectrum in smoking cessation support.

### Implications for future research and practice

The findings of this study illustrate that it would be valuable to raise awareness among professionals about the influence of gender in smoking cessation support. In addition, the findings may help understand the differences in intervention effectiveness and suggest ways to enhance the effectiveness of smoking cessation interventions provided by professionals. Earlier research has shown that interventions that consider the unique needs of women, including those related to sex and gender, are likely to be effective and well-received (Ashley *et al*. 2003, Torchalla *et al*. 2012). For example, it stresses the importance of providing emotional support to women. Emotional support can contribute to a calm and secure interpersonal environment that helps making the difficult task of quitting more achievable (Westmaas *et al*. 2010). On the other hand, assisting men to delve deeper into their feelings and show vulnerability may be beneficial. Reconnecting with and expressing emotions are important tasks as recognizing and expressing fear, shame, and pain play a significant role in the success of recovery for many individuals (Camacho-Ruiz *et al*. 2024). In addition, using a direct communication style may be particularly effective for men trying to quit smoking, as this may resonate more strongly with their preferences, helping the message to come across more effectively (Bottorff *et al*. 2016). While this seems promising, future research should determine how such actions could enhance the effectiveness of cessation support among men and women.

The findings of the study may also help develop tools for professionals to adopt a more gender-responsive approach in smoking cessation support. E-learnings and toolboxes have been developed to guide professionals in implementing gender-responsive strategies which may serve as good examples for developing more specific tools for smoking cessation (AWHA 2024, BMZ 2019). When applied to smoking cessation, such E-learnings could help raise awareness among professionals about the role of client’s gender (e.g. women and men may prefer different communication styles) and about the role of professional’s own gender (e.g. professional women may be perceived as more approachable). More research is needed to explore how the identified differences can be addressed and to determine which strategies are most effective.

## CONCLUSION

This study identified the experiences of professionals providing smoking cessation support, focusing on perceived differences by both clients’ and professionals’ gender. It highlights that gender differences may influence the support provided, suggesting the importance to take gender into consideration when providing smoking cessation support. Further research is needed to determine how addressing these gender differences may enhance intervention effectiveness.

## Supplementary Material

daaf183_Supplementary_Data

## Data Availability

Interview transcripts cannot be shared publicly because of ethical restrictions.
