# Supplementary material for: Gender differences in smoking cessation support: a qualitative study of Dutch healthcare professionals’ experiences
Source: Health Promot Int. 2025 Nov 10;40(6):daaf183. doi: 10.1093/heapro/daaf183 (PMC12599321; doi:10.1093/heapro/daaf183)
Supplement: daaf183_Supplementary_Data [file daaf183_supplementary_data.doc]

**SUPPLEMENTARY MATERIAL 1: INTERVIEW GUIDE**

| **Introduction interview**  **5 minutes** | **Welcoming participant,**  **Introduce yourself as the researcher,**  **Thank them for their time.**  Purpose of the interview  First of all, thank you for your time and participation. The purpose of this interview is to explore the role of gender – both of yours and your client’s – in smoking cessation support from your perspective as a professional.  The interview is structured as follows: we will start with some general introductory questions, then sequentially go through the different stages of the cessation process: ‘consideration’, ‘preparation’, ‘action’, ‘maintenance’, and ‘relapse’. We will conclude with some final questions. During the various phases of the cessation process, I will first ask about the experiences, then the inquire about any gender differences you perceive, the possible challenges you encounter in every phase and ask about the potential role of your own gender.  Overall, this interview will take about 45 to 60 minutes.  Informed consent  With your permission, the interview will be recorded and fully pseudonymized. Some notes will be taken, but these will be handled confidentially. The conversation will be transcribed verbatim and the recording will be deleted. Everything you share during this conversation will be not traceable back to you. This is voluntary and you can stop participating at any time without giving a reason.  Do you have questions?  Do you agree to contribute to the research?  Can you confirm that you consent to the recording of this conversation? |
| --- | --- |
| **Main questions: Context**  **2 minutes** | - To what extent is smoking cessation support part of your job? - What type of support do you offer?   - To what extent is it more individual or group support?   - To what extent is it more general (according to protocol) or tailored to the needs of an individual? - How would you describe the target group you support in smoking cessation?   - Gender   - High/low SEP? |
| **Main questions: Contemplation**  **5 minutes** | - What is your experience with the accessibility of your support?   - How do clients come to you for smoking cessation support?   - To what extent do you see differences between men and women?   - Can you elaborate on this? - To what extent do you experience challenges in the accessibility of the support?   - If yes, what kind of challenges are they?   - To what extent do you see differences between men and women?   - Can you elaborate on this? - To what extent do you notice that your own gender (being a man/woman) influences the accessibility of the support? |
| **Main questions: Preparation**  **5 minutes** | - What is your experience with preparing clients for the support?   - To what extent does your approach differ between men and women?   - Can you elaborate on this? - To what extent do you experience challenges in preparing for the support?   - To what extent do you see differences between men and women in this regard?   - Can you elaborate on this? - To what extent do you consider the needs of the client during the preparation?   - To what extent do you see differences between men and women in this regard?   - Can you elaborate on this? - To what extent do you notice that your own gender (being a man/woman) influences the preparation for the support? |
| **Main questions: Action**  **15 minutes** | - What are your experiences with the progress of the support you provide?   - To what extent do you see differences between men and women in this regard?   - Can you elaborate on this? - What is your experience with involving family and the social environment of the client in the support?   - To what extent does your approach differ between men and women?   - To what extent do you notice that your gender (being a man/woman) influences the involvement of these relationships?   - Can you elaborate on this? - To what extent do you experience challenges in providing smoking cessation support?   - To what extent do you see differences between men and women in this regard?   - Can you elaborate on this? - To what extent do you notice that your own gender (being a man/woman) influences the progress of the support? |
| **Main questions: Maintenance**  **5 minutes** | - To what extent do you experience that clients benefit from your help?   - Can you give examples of elements of the support that in your experience are most/least effective?   - To what extent do you see differences between men and women in this regard?   - Can you elaborate on this? - What is your experience with providing support/follow-up care to clients who maintain smoking?   - To what extent do you see differences between men and women in this regard?   - Can you elaborate on this? - To what extent do you experience challenges in supporting clients in maintaining their behavior?   - To what extent do you see differences between men and women in this regard?   - Can you elaborate on this? - To what extent do you notice that your own gender (being a man/woman) influences the maintenance of the behavior? |
| **Main questions: Relapse**  **5 minutes** | - To what extent do you experience relapses among clients?   *When mentioned in the previous question(s), ignore these questions   - - To what extent do you see differences between men and women in this regard?   - Can you elaborate on this? - What is your approach when a client has a relapse?   - To what extent does this approach differ between men and women? |
| **Closing** | Those were all my questions for you.   - Are there any topics/aspects we have not discussed yet that you find important? - Do you have any additional questions or comments?   Thank you again for your time and for sharing your experiences and perceptions. Your insights are incredibly valuable for this research. Have great day! |
